# “You cannot eat rights”: a qualitative study of views by Zambian HIV-vulnerable women, youth and MSM on human rights as public health tools

**DOI:** 10.1186/s12914-015-0067-6

**Published:** 2015-10-05

**Authors:** Choolwe Muzyamba, Elena Broaddus, Catherine Campbell

**Affiliations:** Independent Researcher, A9, Marshlands Village, PO Box 32379, Lusaka, Zambia; Department of International Health, Johns Hopkins Bloomberg School of Public Health, 615 N. Wolfe Street, Baltimore, MD USA; Department of Social Psychology, London School of Economics and Political Science, St Clement’s Building, Houghton Street, London, WC2A 2AE UK

**Keywords:** Human rights, HIV-Prevention, Key populations, Zambia

## Abstract

**Background:**

Human rights approaches now dominate the HIV prevention landscape across sub-Saharan Africa, yet little is known about how they are viewed by the populations they are designed to serve. Health interventions are most effective when they resonate with the worldviews and interests of target groups. This study examined local Zambian understandings of human rights approaches to HIV-prevention among three highly HIV-vulnerable groups: women, youth, and men-who-have-sex-with-men (MSM).

**Methods:**

Focus groups included 23 women, youth, and MSM who had participated in activities organized by local non-governmental organizations (NGOs) using rights-based approaches, and interviews included 10 Zambian employees of these NGOs. Topics included participants’ experiences and views of the utility of these activities. Thematic analysis mapped out diverse ways participants viewed the concept of human rights in relation to HIV-prevention.

**Results:**

Whilst NGO workers noted the need for human rights programs to address the complex drivers of the HIV epidemic, they struggled to tailor them to the Zambian context due to donor stipulations. Women program beneficiaries noted that the concept of human rights helped challenge harmful sexual practices and domestic abuse, and youth described rights-based approaches as more participatory than previous HIV-prevention efforts. However, they criticized the approach for conflicting with traditional values such as respect for elders and ‘harmonious’ marital relationships. They also critiqued it for threatening the social structures and relationships that they relied on for material survival, and for failing to address issues like poverty and unemployment. In contrast, MSM embraced the rights approach, despite being critical of its overly confrontational implementation.

**Conclusions:**

A rights-based approach seeks to tackle the symbolic drivers of HIV—its undeniable roots in cultural and religious systems of discrimination. Yet, it fails to resonate with youth and women’s own understandings of their needs and priorities due to its neglect of material drivers of HIV such as poverty and unemployment. MSM, who suffer extreme stigma and discrimination, have less to lose and much to gain from an approach that challenges inequitable social systems. Developing effective HIV-prevention strategies requires careful dialogue with vulnerable groups and greater flexibility for context-specific implementation rather than a one-size-fits-all conceptualization of human rights.

## Background

Critical assessments of the ABC-strategy (Abstain, Be-faithful, Condomize) in HIV-prevention in sub-Saharan Africa denounce it for over-simplistically emphasizing individual-level change without accounting for the complex social and structural factors that constrain choice and behavior [1, 2]. Therefore, many public health programs have switched to human rights approaches, integrating human rights norms and principles in the design and implementation of HIV programs [3]. This move is premised on the assumption that social inequalities – particularly relating to gender, age and sexuality – increase the vulnerability of women, youth (15–25) and men-who-have-sex-with-men (MSM) to HIV infection, and reduce the likelihood of effective service access and treatment adherence by People Living with HIV [4, 5].

Zambia is one of the countries in Sub-Saharan Africa most hard hit by the HIV/AIDS epidemic, with 14 % of all adults ages 15–49 infected and nearly one in five children orphaned [6]. An analysis of demographic and health survey data between 2003 and 2007 indicates no statistically significant decline in country-wide prevalence [7]. Overall, HIV infection rates among women are 25 % higher than among men—and this disparity is particularly pronounced in youth ages 20–24 in which female prevalence, at 12 %, is over twice that of males [6]. A recent study of antenatal clinic surveillance data indicated that HIV prevalence among teenage girls had declined to around 8 % in 2008 from a high of 14 % in 1994, but those declines stagnated between 2008 and 2011 [8].

Among adults in Zambia, 90 % of HIV infection occurs through heterosexual activity, with key drivers of the epidemic described as including multiple and concurrent partners, low and inconsistent condom use, and mobility and labor migration [9]. Further examination reveals a complex interplay of economic deprivation, gender inequality, discrimination, and lack of educational and employment opportunities that increase vulnerability and thus facilitate expansion of the epidemic. As of 2010, over sixty percent of the Zambian population lived in poverty [10]. Income inequality is profound, with 70 % of the Zambian people sharing only 20 % of income [11]. Unemployment averages around 16 %, but is particularly pronounced among young people aged 15–35 where it reaches over 27 % [12]. Although improvements in Zambia’s Human Development Index meant that it was recently moved into the Medium Development Category, on the Gender Inequality Index it was still ranked 135 out of 148 countries. This places it below many countries in the Low Development category and below the majority of its neighbors in sub-Saharan Africa [13].

Exacerbating their vulnerability to the widespread poverty and unemployment described above, Zambian women face daunting social and economic inequality. Only 25 % have a secondary or higher level of education compared to 44 % of men, and only 73 % of women participate in the labor market compared to 86 % of men [13]. Additionally, men are far more likely to be engaged in wage labor than women, who work primarily in non-wage agricultural roles [12]. Although women generally do the majority of the labor in agricultural households, they have less access to land, markets, credit, and agricultural inputs [14]. These disparities are compounded by social systems that define women’s identities primarily in relation to the men in their lives, strict gender roles dictating women’s subordination to their husbands, and a legal system that codifies discriminatory resource distribution [14, 15]. For example, the customary laws that prevail in the local courts of rural areas define a bride as the property of her husband and his family after marriage [14]. This means she has no rights to property, finances, or custody of children if the marriage ends. As a result, women are often entirely reliant on their husbands for both economic support and social identity. Half of all Zambian women reported experiencing domestic violence, one-third of them in the past year [6]. Women also have little power to negotiate the terms of or refuse sex, leaving them vulnerable to transmission of HIV from husbands with multiple sexual partners [16, 17].

Similarly, Zambia has a large youth cohort—66 % of the population is 24 or younger—that struggles with employment and access to education opportunities. In rural areas, the vast majority of youth work in agriculture with few chances to escape poverty, while in urban areas 41 % of girls and 24 % of boys were neither employed nor enrolled in school [12]. The legacy of colonialism and more recent neoliberal structural adjustment policies have left Zambia with an underfunded education system poorly equipped to prepare young people for the modern economy or to critique and challenge the inequities they face [18]. Many aspire to work in the public sector, or in skilled jobs within the private sector, yet obtaining the skills needed for these positions through higher education is a reality for only the richest members of society. Thus many youth turn to transactional sex or drug dealing to make a living, or have little choice other than non-wage agricultural work [19].

“Key populations,” such as MSM, make up roughly 10 % of new HIV infections in Zambia and are a critical area of focus for prevention efforts, yet discrimination against such populations can impede these efforts [9]. Due to the intense stigma that they face, little data exists on the size and health status of Zambia’s MSM population, and across sub-Saharan Africa epidemiological data for MSM is poor [20]. However, a recent study estimated the HIV prevalence rate among MSM in Zambia at 33 %, one of the highest in Southern Africa [21]. The MSM population has been greatly overlooked in the country’s HIV prevention efforts, with policy-makers and even some of those working in the HIV prevention field sometimes denying that male-to-male sex occurs in Zambia [22]. MSM face intense social and religious discrimination, and politicians and activists working to advance gay rights encounter vitriolic accusations of being anti-Christian and un-Zambian [23].

Amidst this context of social and economic inequities that marginalize and increase the vulnerability of women, youth, and MSM, the human rights approach has been embraced by international funders, the Zambian government, and local non-governmental organizations (NGOs) [24]. Through the National AIDS Council (a branch of the Zambian ministry of health), NGOs are encouraged to implement programs that support human rights principles when designing, implementing and monitoring their activities. NGOs comply by implementing advocacy programs aimed at securing human rights enjoyment and protection for those most vulnerable to HIV. As of now, there have been few efforts to promote rights by incorporating or collaborating with local institutions such as churches, cultural organizations, or the legal system. All current emphasis is on reaching out to vulnerable groups directly.

Zambian NGOs that take a rights-based approach encourage participation through various means including music, films, drama, poetry and art competitions, poems, door-to-door discussions, magazines, radio and TV talk shows, billboards, community outreach programs, workshops and development of safe spaces like youth friendly corners [25]. Such activities aim to give beneficiaries the urgency to challenge factors preventing them from enjoying and claiming their rights. Organizations emphasize sexual and reproductive health rights such as having the power to negotiate terms of sexual activity. They also work to counter stigma and discrimination, and promote messages regarding equality of status for all in society regardless of age, gender, or sexual orientation [25, 26].

The underlying assumption is that members of socially marginalised groups, such as youth, women or MSM, are most likely to change health-damaging behaviours on the basis of intertwined processes of participation and partnership. Participation in collective activities—from peer education to community-strengthening programs –serves as a pathway to the development of health-enhancing insight and solidarity. This occurs through the development of a common awareness of the rootedness of health problems in shared experiences of oppression and a determination to overcome these [27, 28]. The potential for effective participation depends heavily on the extent to which participation by marginalised groups is backed up by the development of partnerships with powerful agencies [29, 30]. Institutions such as local and global NGOs and public sector health, welfare and other relevant ministries have the power to create policies, services and interventions that support the possibility of more health-enhancing behaviours among the marginalized.

Human rights strategies for social development and public health have received mixed reactions in Africa. Proponents argue that at the structural level a human rights approach can be useful for holding states accountable and binding them to action and enactment of policy. Such policy can secure access to entitlements like health care and education [4], and address long-standing inequities codified in both social systems and at the policy level that increase vulnerability to HIV [15]. Yet critics assert that in practice human rights efforts often shift focus towards individual civil and political rights at the expense of social and economic rights and structural barriers, thus neglecting critical issues like poverty and inadequate service provision [31, 32]. Others call into question the ahistorical and decontextualized nature of the dominant human rights discourse and its claims for a universal morality. They argue that in fact it furthers a neo-colonial imposition of Western ideals and norms onto non-Western cultures [33, 34]. Mutua describes it as a “Eurocentric colonial project, in which actors are cast into superior and subordinate positions (p.204).” Jensen and Gaie suggest that in sub-Saharan Africa, human rights approaches to HIV prevention in their current form conflict with the ‘socio-ethical’ ideals of African communalism [35]. They assert that instead a revival of indigenous understandings of personhood is needed.

Despite this ample theoretical literature, only a handful of studies, described below, have examined the use of a human-rights platform for HIV-related efforts in practice in sub-Saharan Africa. In their case study of a community mobilization project in South Africa with a gender empowerment agenda, Campbell and Nair [36] noted that women clearly prioritized economic concerns over rights and saw challenging male power as dangerous and counter-productive. Similarly, Mannell [37] found that gender awareness, as it was framed by international donors, was not seen as a priority by community-based organizations in Malawi and Zambia, but rather as a foreign and un-African concept imposed by outsiders. More positively, in a recent qualitative study with Zambian adolescents living with HIV, respondents embraced the concept of human rights, particularly those related to confidentiality, control over disclosure, and access to reliable information and care [24]. Clearly, views of rights differ dramatically in perhaps unexpected ways. Yet no research has been done regarding local understandings of human rights approaches for HIV prevention, or exploring how the perspectives of vulnerable groups may differ within the same context.

Despite its current popularity and continued application, little is known about how effective a human rights approach is in inspiring behavior change, and to what extent it is appropriate to tackling the drivers of HIV-related social inequality in in Zambia’s settings, and in sub-Saharan Africa more broadly [36]. Understanding how specific sub-populations interpret and experience a given HIV intervention is essential for optimizing program effectiveness as health interventions tend to be most effective when they resonate with the worldviews and perceived interests of target groups [28]. Against this backdrop this study explores the ways in which the three most HIV vulnerable groups understand the concept of human rights. Our aim is not to evaluate the impact of the rights-based approach, but rather to document and examine these local understandings. This paper seeks to advance knowledge in this area through a case study from Zambia, where as yet no research has explored the social appropriateness of the human rights approaches that dominate the HIV response.

We locate our study against the background of Social Representations Theory (SRT), a social psychological framework for investigating how particular social groups make sense of new and often unfamiliar social phenomena. Social representations are understood as the conceptual tools people use to give meaning to their everyday lives, with collectively negotiated social knowledge viewed as the property of groups rather than individuals. Social representations help in establishing a shared social reality [38]. This shared social reality is particularly important in public health efforts because people’s perspectives and shared social understandings of HIV interventions help to frame their reaction towards public health efforts [28]. SRT acknowledges that socially negotiated knowledge is continually contested and debated as groups (be they women, youth or MSM) go about their everyday lives. The social representations approach thus aims to map out different ways available to members of a social group for making sense of a social phenomenon, such as human rights. It does not seek to suggest that all individual group members think in the same way, or to generate stereotypical accounts of how ‘a typical group member’ thinks. In this study, SRT served as a heuristics device, guiding our analysis and presentation of results by directing attention to the varied and at time conflicting aspects of the information provided by our respondents.

## Methods

We conducted a qualitative study that collected data at one time-point and subjected findings to interpretive thematic analysis [39]. In designing the study and conducting the analysis we adhered to the BioMed Central qualitative research guidelines for relevance, appropriateness, transparency, and soundness (RATS). Data consisted of focus group discussions (FGDs) with 23 respondents and interviews with an additional 10 respondents, for a total of 33 study participants. FGDs were conducted with the beneficiaries of projects related to human rights and HIV. The three focus groups included 8 youth, 8 women, and 7 MSM, conducted separately by respondent group. The focus group with youth included both male and female adolescents to increase the diversity of opinions expressed and to provide opportunities for a wider and more varied discussion. The decision to use a mixed-gender composition was also informed by a review of literature on research with vulnerable groups that indicated its acceptability given the nature of the topics of interest [40, 41]. In-depth interviews were conducted with 10 local staff members of NGOs that serve women, youth, or MSM. Participant socio-demographic information is displayed in Table 1.

**Table 1:** Socio-demographic information for NGO workers, youth, women, and men-who-have-sex-with-men (msm) participants

Participant number	Gender	Age	Education	Employment
NGO worker interview participants				
1	Male	Mid-30’s	Beyond High School	Youth NGO Staff Member
2	Male	Late 20’s	Beyond High School	Youth NGO Staff Member
3	Female	Mid-20’s	Beyond High School	Youth NGO Staff Member
4	Female	Late 20’s	Beyond High School	Women’s NGO Staff Member
5	Female	Early 30’s	Beyond High School	Women’s NGO Staff Member
6	Female	Mid 20’s	Beyond High School	Women’s NGO Staff Member
7	Male	Early 30’s	Beyond High School	MSM NGO Staff Member
8	Male	Early 30’s	High School	MSM NGO Staff Member
9	Male	Late 20’s	Beyond High School	MSM NGO Staff Member
10	Female	Early 30’s	Beyond High School	MSM NGO Staff Member
Youth FGD participants				
1	Female	Early 20’s	Beyond High School	Student
2	Male	Early 20’s	High School	Unemployed
3	Female	Early 20’s	High School	Unemployed
4	Male	Early 20’s	Beyond High School	Employed
5	Male	Late Teens	High School	Unemployed
6	Female	Early 20’s	High School	Student
7	Male	Early 20’s	Beyond High School	Employed
8	Female	Late Teens	High School	Student
Women’s FGD participants				
1	Female	Early 30’s	High School	Employed
2	Female	Late 20’s	Beyond High School	Employed
3	Female	Late 30’s	Elementary	Unemployed
4	Female	Early 30’s	Beyond High School	Employed
5	Female	Early 30’s	High School	Unemployed
6	Female	Early 20’s	Elementary	Unemployed
7	Female	Mid-20’s	High School	Employed
8	Female	Early 20’s	Elementary	Unemployed
MSM FGD participants				
1	Male	Mid-30’s	Beyond High School	Employed
2	Male	Early 20’s	High School	Employed
3	Male	Early 30’s	Beyond High School	Employed
4	Male	Late 20’s	High School	Employed
5	Male	Mid-20’s	Elementary	Unemployed
6	Male	Mid-20’s	High School	Employed
7	Male	Late Teens	High School	Employed

The first author, a native Zambian with experience working in the area of HIV-prevention, established collaborations with three Lusaka-based NGOs for the purposes of this study. While there are many NGOs that work with vulnerable groups in Lusaka on HIV-prevention, those included in this study were selected based on which populations they served and to what extent a rights-based approach was operationalized in their missions and activities. The three organizations selected for collaboration were those that the first author, based on his previous experience working with numerous Lusaka-based organizations, identified as taking the most extensive rights-based approach to HIV prevention and that were engaged in comprehensive outreach and education efforts targeting one of the vulnerable populations of interest. NGO staff facilitated recruitment of FGD participants by inviting beneficiaries who indicated availability and willingness to share their perspective, and who varied in age, educational level, and occupation-type. NGO employees were selected for interviews based on the relevance of their duties to the study’s research objectives—we recruited staff that were directly involved in service delivery and advocacy efforts with the population of interest, and/or played a role in program planning and determining organization strategies.

FGDs averaged 90 min and interviews averaged 40 min. All were conducted by the first author,^1^ following a topic guide but allowing for discussion of emergent areas of interest to the participants. The FGD topic guide included 12 broad questions regarding beneficiaries’ experience with rights-based HIV prevention activities, their thoughts on how beneficial and suitable such activities were, the aspects that they liked and disliked, and recommendations for change. The topic guide used to interview NGO employees consisted of two to three questions on each of four broad areas: organization activities, experiences with the human rights approach, reasons for taking a human rights approach, and challenges encountered. During both FGDs and interviews, follow-up questions were also asked for clarification, and to encourage respondents to expand upon their answers. Participants came from numerous ethnic groups and thus spoke different local dialects; however all spoke at least basic English, Zambia’s official language. All FGDs and interviews were therefore conducted in English to ensure consistency and ease of discussion amongst respondents. However, participants were also encouraged to express themselves freely in their ethnic dialect as needed. Ethnic words and phrases used were later translated into English during the transcription process, or left un-translated if there was no good English equivalent.

All FGDs and interviews were tape recorded, transcribed and coded by hand, also by the first author. Thematic analysis was guided by Social Representations Theory (SRT), and involved the repeated reading and re-reading of transcripts, to identify basic codes, which were progressively clustered in 15 organizing themes and 8 global themes (see Table 2). SRT emphasizes the contested nature of social representations. This led us to look for ways that rights were positively and negatively represented, and to look for similarities and differences in these representations both within and across groups.

**Table 2:** Coding framework

Respondent group	Global theme	Organizing theme	Basic themes
NGO Workers	NGO Workers’ Positive Representations of the Human Rights Approach	Effective Strategy for HIV Prevention	• Necessary for addressing the complex drivers of the HIV epidemic
			• Raises awareness about discrimination and inequality
		Means of accessing resources	• Way of getting support and funds from donors
	NGO Workers’ Negative Representations of the Human Rights Approach	Conflicts with Cultural and Religious Values	• Lack of flexibility in implementation due to need to conform to donor agency agendas
			• Messages viewed as encouraging disrespect of parents and elders
		Structural Limitations to Effectiveness	• Weak and ineffective legal system
Youth	Youth’s Positive Representations of the Human Rights Approach	Engaging Activities Used to Disseminate Messages	• Interesting and enjoyable concerts, dramas, and other events
			• Opportunities to gain information on HIV and sexual health
		Participatory in Nature	• Opportunities to interact and discuss issues with other youth
			• Consideration for youth’s opinions, rather than top-down behavioral proscriptions from “experts”
	Youth’s Negative Representations of the Human Rights Approach	Conflict with Cultural and Religious Values	• Messages viewed as encouraging disrespect of parents and elders
			• Human rights as an “un-Zambian” Western concept
		Failure to Address Youth’s Priorities and Day-to-Day Realities	• Dependence on family structures for material support and survival
			• Lack of job and education opportunities
			• Poverty and unemployment as risk factors for HIV
Women	Women’s Positive Representations of the Human Rights Approach	Basis for Challenging Oppression and Mistreatment	• Opportunities to discuss previously taboo topics regarding harmful traditional practices
			• Grounds for objecting to harmful sexual practices
			• Protection from domestic violence and abuse
	Women’s Negative Representations of the Human Rights Approach	Conflict with Cultural and Religious Values	• Messages viewed as encouraging women to defy their husbands and disrupt marital harmony
			• Human rights as an “un-Zambian” Western concept
		Failure to Address Women’s Priorities and Day-to-Day Realities	• Dependence on husband for material support and survival
			• Disconnect with unmarried human rights champions
			• Poverty and unemployment as risk factors for HIV
Men-Who-Have-Sex-With-Men (MSM)	MSM’s Positive Representations of the Human Rights Approach	Promoting Urgently Needed Change	• Current environment of extreme discrimination and lack of rights
			• Traditional religious and cultural values that stigmatize homosexuality
		Platform for Challenging Discrimination	• Human rights approach used to promote equality
			• Human rights approach used to expose abuses
		Effective Community Mobilization	• Bringing together the MSM community to advocate for rights
			• Creation of support networks and safe spaces
	MSM’s Negative Representations of the Human Rights Approach	Challenges Limiting Effectiveness	• Weak legal system incapable of defending rights
			• Inadequate funding
			• Implementation of approach done in a way that is too confrontational with traditional cultural and religious values

The study was approved by the ethics committee at the London School of Economics. Because of the non-clinical and non-invasive nature of the study, it did not require review by a Zambian health research ethics board. Given the complex ethical issues that arise when conducting research in resource-limited nations [42], extensive care was taken to ensure that data was collected ethically with sensitivity to the local context. The first author, a native Zambian, conducted all interviews and FGDs himself, first ensuring that participants were fully informed about the details of the study and that their participation was completely voluntary, and then obtaining written informed consent. He was the sole member of the research team with access to recordings, and shared transcripts of NGO workers’ interviews and women’s and youth’s FGDs with the rest of the team only after de-identification. Due to the intense stigma against homosexual individuals in Zambia, additional steps to ensure the anonymity of MSM respondents were taken on top of the protocols already in place for maintaining confidentiality and respecting respondent privacy. Only the first author had access to the data from MSM respondents—he shared only de-identified quotation segments (rather than the full transcripts) with the other authors during analysis and manuscript writing. In addition, in the results reported below, we take care not to reveal the names of the organizations whose employees and beneficiaries we recruited, or to disclose any information that might make the organizations or individual respondents identifiable.

## Results

### NGO workers

To situate the perspectives of our youth, women, and MSM respondents within the context of rights-based HIV-prevention initiatives in Zambia, Zambian staff members from three different local NGOs that serve vulnerable populations were asked to describe the reasons that their organization took a rights-based approach, and the challenges encountered. Although representations of the human rights approach varied, we noted no major differences between participants related to whether their organization served women, youth, or MSM. They described a variety of reasons that their organizations utilized a rights-based approach regarding its contributions to their overall objectives of preventing HIV and promoting the health of vulnerable groups. For example, respondents noted that an emphasis on rights was necessary for addressing the complex drivers of the HIV epidemic and proved effective for raising awareness about discrimination and inequality:

*“After working in the area of advocacy for a long time, giving people information about HIV, I realized that there was a missing link. This is because no matter how much information you dish out to people it cannot help them if they cannot enjoy their basic human rights. The issue of rights became so clear to me as one of the major impediments in the process of eradicating HIV among young people in Zambia… There are a lot of traditional practices that are performed on young people against their wishes just to fulfill tradition and our role has been to go there and challenge these practices… Young people are abused sexually and merely giving them information about HIV without tackling the abuses of their rights is counterproductive.”*

*-Interview participant 3, female, mid-20’s, staff member at an NGO working with youth*

*“We have really achieved a great deal of success… We have managed to raise awareness on the silences patterning MSM, we have managed to highlight the lack of legal support for people with different sexual orientation. We have raised the discussions and it is only a matter of time that people will start seeing our results. Of course it is not that easy but we have begun challenging these complexities that hinder people from expressing their sexuality freely.”*

*-Interview participant 7, male, early 30’s, staff member at an NGO working with MSM*

NGO workers also talked about more pragmatic reasons for taking a rights-based approach, emphasizing the importance of aligning themselves with donor organizations’ interests in order to access the funding needed to sustain their organizations. Some framed this in positive terms, explaining that incorporating an emphasis on human rights into their programs had helped them to leverage additional resources and funding and had facilitated beneficial relationships with large international organizations. However, the need to align with donors’ rights-promotion agendas was also often spoken of negatively. Several respondents explained that adopting a rights-based approach was basically compulsory, and limited their organization’s programming options:

*“We work in line with what most funders suggest in our quest to reduce HIV… International funders have prioritized the use of this approach in an effort to push their agendas. These days without a human rights approach you will not access funding. It is all about funding… That is why you find most NGOs these days are working on human rights issues. Although I have found the approach to tackle human rights useful, sometimes I just wish organizations had more flexibility in the manner in which they use the money. Because we, the NGOs on the ground, [are the ones] who know our people better. So sometimes you might want to do things in a different way but funders do not like that… We need to have more say in how we run our programs… We Zambian NGOs know our people more, we know our culture more. So in that sense, it defies all logic to rely on western people to map out our interventional programs without even having a clue of how the environment is.”*

*-Interview participant 4, female, late 20’s, staff member at an NGO working with women*

As the quote above illustrates, respondents expressed frustration with the lack of flexibility to tailor their activities and approach as they saw fit. This was often discussed in tandem with descriptions of resistance encountered from community elders and leaders to the messages disseminated by rights-based programs. NGO workers explained that human rights, as they were framed by these programs, were seen as threatening to traditional values and social hierarchies:

*“While this approach seems appealing, it invokes retaliation from elders and leaders in the communities. It is a situation where it seems their authority is being challenged. It can’t work like this you know. Elders in society are very instrumental in the process of change but when they feel their authority is being challenged they will respond by frustrating your efforts and your entire project ultimately fails. So sometimes we actually prefer having a more involving approach that does not result in contestation but some funders do not see this… So it is a struggle when are find ourselves at such dead ends. We are the ones who need the funding so we just have to switch our approach to suit the funders because without their money we cannot do much.”*

*-Interview participant 2, male, late 20’s, staff member at an NGO working with youth*

Finally, NGO employees also noted the lack of a strong legal system as a significant structural barrier to the human rights approach. They explained that while their programs and messaging urged beneficiaries to know and advocate for their rights, often these rights were not recognized legally. Even those rights that were technically written into law, for example prohibitions against physical and sexual abuse of women and children, were often not enforced. NGO employees said that without legal backing, messages about rights were unconvincing, and described a political and legal environment where there was little actual commitment to creating and enforcing human rights protections:

*“It has been extremely difficult to overcome the challenge of insufficient legal framework… The institutions in this country are also very weak… We sometimes feel we have no one in this country to rely on. They all just talk the talk and never walk the walk. The politicians always talk about how important it is to promote human rights but that is where it ends, it’s all lip service.”*

*-Interview participant 8, male, early 30’s, staff member at an NGO working with MSM*

### Youth

Youth respondents all reported experiencing and being active participants in rights-based HIV-prevention activities run by a local NGO with a specific focus on youth. When youth were asked to describe these activities they at first praised the interactive strategies that the NGO used to disseminate messages, such as drama, concerts, and “youth friendly corners” at hospitals. They found such activities engaging and entertaining and appreciated the opportunity to obtain new information and interact with other young people.

*“The youth friendly corner in Kanyama [hospital] has been helpful for me, for example we go there to engage with other young people who are facing similar things like me, get information about how to help fellow young people who have been sexually abused.”*

*-Youth FGD participant 5, Male, late teens, unemployed*

Several respondents also noted the participatory nature of these activities, saying that they were encouraged to develop and voice their own opinions, which contrasted sharply with the programming implemented during the ABC era:

*“In the ABC people would just come and command us to abstain. But these guys I feel involve us a bit more… At least I can say that we participate in activities and not just going there to listen to experts say what they have to say and we on the other hand are just supposed to follow whatever has been said by the so called experts.”*

*-Youth FGD participant 4, male, early 20’s, employed*

Yet while the youth praised the strategies and mediums through which local NGOs disseminated information regarding rights of young people, the messages themselves they described as incompatible and corrosive to their cultural and religious values:

*“They have a lot of these activities that are just so interesting and I go there to take part… It is always an awesome experience to interact with youths from different backgrounds… I contribute whenever possible and especially if I agree with the message being preached… Because some of the messages are just not conducive for a person like me if you know what I mean. You know they in some ways encourage young people to disobey their parents. I find that unbearable… We have all been brought up to respect our fathers and mothers. But their activities sometimes seem to encourage the very opposite. So when they begin talking about such things I just shut off and stop listening. But don’t get me wrong, there are some times when they really talk about very helpful things about HIV. But in general when they begin their talks on disrespecting parents people just feel uncomfortable you know. It is not part of us, it is not in us. It is unZambian.”*

*-Youth FGD participant 1, female, early 20’s, student*

*We learn from the scriptures that anyone who is older than you deserves to be respected. Now, this idea of challenging them is contradicting the spirit of our religion… Given a choice to choose between rights and my God, I will definitely choose my God any day any time…*

*-Youth FGD participant 3, Female, early 20’s, unemployed*

In addition to the ways that the human rights approach was perceived to negate the respectful and deferent relationships between youth and their elders called for by cultural and religious values, our youth respondents’ comments also indicated how it threatened the material obligations implied by these relationships, which they relied upon for their sustenance.

*We need to be vigilant to preserve what is ours. This is not in our culture. And we have to be very candid about that. This is a western phenomenon. We have certain cultural codes that we live by. These are being threatened by these same human rights we are talking about. They are making us fight against the people who feed us, take care of us and stuff like that. Everything I have on me has been provided by my parents and it is an abomination to stand against them in the name of rights. Rights that are not even tangible. You cannot eat rights when your father chases you away from his home, can you? These youths from [the NGO] have jobs and have an income, yet they expect me to stand against my father. Where will I go when he kicks me out of the house? Our positions are different. Impossible!*

*-Youth FGD participant 7, male, early 20’s, employed*

Of the eight respondents in the youth FGD, the majority had only a high school level education and none had full-time employment. In contrast, most young NGO employees who championed the human rights approach held university degrees and were paid well for their positions. They were therefore described as out of touch with the day-to-day educational and economic challenges of the majority of Zambian youth.

*“Most of these youths working for YVZ have jobs, they have gone to school. So they can manage to stand on their own. But what about me who has not been to college, has no job and is only surviving because my father is keeping me. What should I do? Disobey my father? …We cannot be following what they say when we have different situations in life. We are poor, they are richer.”*

*-Youth FGD participant 6, female, early 20’s, student*

Youth respondents unequivocally situated rights as secondary to material issues in terms of both their own priorities and regarding risk factors for HIV. All youth respondents talked extensively about Zambian young people’s unmet needs for education and employment opportunities and described these as much more pressing concerns than recognition of their rights. Several also traced out the connection between poverty and vulnerability to HIV, expressing their opinion that ensuring access to basic necessities like food and healthcare should be prioritized over human rights:

*“I think it is more important to find jobs before spending most of our time discussing these issues…Why not talk about the many important challenges that we face? Like lack of education, jobs and such things… I think the number one problem is poverty and not really rights.”*

*-Youth FGD participant 3, female, early 20’s, unemployed*

*“Let us make sure that our people have food on the table, good access to health services before we can start talking more cosmetic issues like human rights… Just look at how young girls are selling their bodies so they can make some money. It is this that puts them at risk of contracting HIV. It is poverty, lack of education and jobs that send them to the streets to sleep with different men so they can have food on the table. This is more critical. Human rights cannot be talked about before poverty eradication.”*

*-Youth FGD participant 2, male, early 20’s, unemployed*

Although youth respondents emphasized the critical nature of the work that NGOs were doing to address the HIV epidemic and expressed their approval for the overall objectives of these organizations, they called for a shift away from the rights-based approach. They asserted the need for HIV prevention workers to engage in dialogue with and incorporate the priorities and ideas of the young people that they were trying to serve. As Participant 4 said, “We need indigenous solutions. We need Zambian solutions.”

### Women

During the discussion women were asked to describe their experiences with the activities of a local NGO working to promote HIV prevention by increasing awareness about women’s rights, as well as what they thought about this approach. Like the youth respondents the women described varied representations of the rights-based approach, noting some positive changes associated with it, but primarily critiquing it fiercely for clashing with cultural values and being out-of-sync with their own priorities.

On the positive side, some women felt that the rights approach had given them grounds to challenge male domination in the community, and that the NGO provided some protection against domestic violence, sexual harassment, and discrimination. The women also noted that it brought previously taboo discussions about sexual health and sexual practices into the public domain. Respondents explained that this increased openness gave women greater power to challenge practices such as early marriage, “*kupyana,*^2^” and forced intercourse that can cause physical and psychological harm, and put women at risk of contracting HIV:

*“Today we have an opportunity to demand for better services for the women in the community because of [the NGO]… Look, we used to have a lot of men marrying off their daughters at a very young age, today they are scared to do that because [the NGO] will speak out and they can be arrested… We are free talking about sex matters and other issues which were previously considered to be taboo… We are freely discussing this because GA has provided a platform to do so… through this program women are free to complain about the use of herbs and rape by husbands. Kupyana (sexual-cleansing) has now become a thing of the past, women can refuse to under-go such practices”.*

*-Women’s FGD participant 6, early 20’s, unemployed*

Yet the vast majority of the discussion focused on the inappropriateness of the human rights approach. Like youth, women described the human rights approach as incompatible with cultural and religious values due to the emphasis on challenging traditional authority hierarchies, which for women centered on marital roles and relationships.

*“You have to realize that their activities have not been successful because they are conflicting with people’s views on culture and religion in the community… Let these people realize that women in Zambia have certain priorities and beliefs which they need to play by, without which they will be wasting their time. I am telling you. I have seen such things before. You cannot just go into some community and assume people there are dull and stupid and you are the only one who can make their situation better with your mzungu ideas.”*

*-Women’s FGD participant 5, early 20’s, unemployed*

These women’s comments indicate a deep connection between cultural identity and defined gender roles, and demonstrate a sense of threat from and animosity towards disruptive ideas imposed by outsiders, harkening back to colonialism:

*“My parents always reminded me of how important preserving our culture is. You know our parents grew up during the colonial times so they more than anyone else understand the importance of preserving culture. These NGOs come in our communities and tell us that times have changed we need to adopt new ways, in short, they tell us to adopt mzungu culture. When growing up, a girl is taught on how to take care of family. Your husband is the most important person in your life as a woman. So whatever happens you need to ensure your husband is well served. Now, look at what these NGOs are telling us to do. They are telling us that we have the right to deny our husbands sex if we so wish. What kind of African woman does that? That is taboo. And these are the things that these people are telling us.”*

*-Women’s FGD participant 3, mid-30’s, unemployed*

Women respondents also noted the vast differences between themselves and the often single or divorced NGO workers who promoted the concept of rights, arguing that these unmarried and educated women had a poor understanding of the issues and priorities of most Zambian women:

*“Look at all these women claiming to be champions of rights. Most of them are not even married. They have broken homes in short. So how do you expect them to really understand our issues as married women?”*

*-Women’s FGD participant 1, early 30’s, employed*

Women respondents emphasized the importance that women in the community placed on having harmonious marriages with their husbands, contrasting this priority with messages disseminated by NGOs that encouraged them to challenge male domination and domestic violence. Efforts to compel them to challenge husbands, whom they depended on for economic survival, were described as outrageous:

*“Most of us have children that we need to take care of and by standing to fight against the man who provides for me and my kids just makes me look stupid. I think for me, what is important is to have peace in marriage, and I see this whole feminist talk as a threat to the peace in my marriage. I do not work, my husband has been taking care of me for 11 years, it is foolish for me to then walk up to him and start talking about my rights. It’s not even fair when you look at it you know. How does that even show any form of gratitude to someone who has seen you through all the difficulties.”*

*-Women’s FGD participant 5, early 30’s, unemployed*

*“I sometimes feel there is a misunderstanding on the part of [the NGO] on what the real issues are. Because on gender based violence they have been constantly telling us how we need to report our husbands to the police from what they term as gender based violence so that our husbands can be arrested. I find this to be highly illogical. So we take the people taking care of us as well as our children to the police and then when they get imprisoned who will look after us? That’s how disorganized their message is. There is no regard for the women afterwards.” -Women’s FGD participant 7, mid-20’s, employed*

Several women very clearly articulated the failure of the rights-based approach to address material factors like poverty and unemployment. They spoke at length about the salience of day-to-day challenges in ensuring access to necessities such as food, water and shelter. Respondents identified these material concerns as the real priorities for women in their community, noting that it seemed unrealistic and foolish to be concerned about rights when basic needs often went unmet:

*“The society has so many problems and it is sometimes better to concentrate on poverty, unemployment among these women in the community [rather] than concentrating so much on rights which in my eyes are not the biggest problem. Look, we do not have proper running water in this place; electricity is a rare commodity for most of the people.” -Women’s FGD participant 2, late 20’s, employed*

Women also noted the key role that economic insecurity and material deprivation played as risk factors for HIV. They explained that lack of employment options caused single women to be exposed to HIV when they were forced to rely on sex work to support themselves. They critiqued the emphasis on human-rights, saying that it neglected these core material factors that increased vulnerability to HIV, and threatened to undermine the relationships most women relied on for basic necessities:

*“I do realize the effects of HIV. But, if I stand up against my husband, he will chase me from his house, then I will become poor and what will I do to make ends meet? I will join those ladies who stand in the streets in the night to sell their bodies so that they can have food. This to me increases the chances of getting HIV more than having me tolerate a husband who once in a while slaps me. The real cause of high HIV rates in women is poverty. This is why we have so many young ladies selling their bodies as sex workers. This rights talk is very superficial. There are deep-rooted problems in this country which have subjected women to so much destitution and rights for me are last on the list. We need to be clear on that. But that is not to say that the message on rights is completely useless. I am just saying that it is being misapplied.” -Women’s FGD participant 1, early 30’s, employed*

Finally, like youth, women also called emphatically for locally informed HIV-prevention approaches. Many described rights-based programs as promoting western concepts that did not fit well in Zambia. They described the need for HIV-prevention efforts tailored to the reality and world-view of Zambian women, rather than those developed by foreigners:

*“It is time for us to fight HIV in a more logical way without adopting western cultural norms and in the process abandoning what is ours. We need indigenous solutions. Solutions that fit in our culture and religion. Without having such a strategy we will continue experimenting on mzungu strategies, in the process losing our identity as a people.”*

*-Women’s FGD participant 3, mid-30’s, unemployed*

### MSM

Unlike the women and youth respondents, MSM were overwhelmingly positive about the human rights approach. As described above, women and youth noted positive aspects but primarily critiqued the rights approach as disruptive to traditional social structures and religious values that they relied on for identity and material support. In contrast, MSM talked at length about the discrimination and stigma that they faced *due to* traditional social and religious values:

*“In this country we are still facing unnecessary rejection from society. People feel we are here to obliterate their culture, yet we are just living our lives in peace and harmony. It is them that look down on us, call us different names on the streets. They can’t even allow us in their churches yet they preach inclusiveness and love. There is just too much bigotry in this country.” -MSM FGD participant 4, late 20’s, employed*

*“There are still a lot of challenges that need to be addressed by especially the Government which has shown no support whatsoever because it is full of Christians and moralists who see us as being immoral in a way. I want to live in a country where I will receive equal treatment and will not be looked down upon as some kind of a deviant in society. I want my rights to be respected, even though some people do not agree with me. I am as human as they are, so why should my case be different?”*

*-MSM FGD participant 7, late teens, employed*

The MSM respondents also noted ways in which discrimination impaired care-seeking and made them hesitant to disclose their sexuality to service providers:

*“I am sometimes skeptical about disclosing my sexuality at the clinic when I go because if I do, the nurse will look at me as a terrible sinner who needs no mercy. You know how religious these people are and anything that has to do with homosexuality is stench from hell.”*

*-MSM FGD participant 2, early 20’s, employed*

Against this backdrop of intense stigma, MSM, desperate for change, venerated the rights-based approach and demonstrated urgency for change, explaining that their rights had been utterly neglected prior to human rights programs. They described it as useful for promoting campaigns on equality, and fighting stigma and discrimination. They viewed it as an effective tool not only for challenging factors that predisposed them to HIV, but also for exposing and slowly working to reduce abuses based on sexual orientation and gender identity more broadly, such as psychological distress of social stigma, unequal treatment, discrimination in jobs and housing, and denial of recognition:

*“I feel we have come a long way and these campaigns have been of great help. They have helped to expose some of our sufferings and though most people in this country do not yet agree with the message, slowly some hearts are being won. Rome was not built in one day, so slowly but surely, the message is sinking down. We should not be so naïve to expect 180 degrees turnaround in attitudes within 5 or 10 years. It will take a bit longer but it’s good that the discussion has started.”*

*-MSM FGD participant 6, mid-20’s employed*

*“You can now see how intense campaigns for equal rights have taken center stage in our country. You couldn’t hear of such things back in the days.”*

*-MSM FGD participant 7, late teens, employed*

Moreover, many MSM respondents credited the rights-based approach with mobilizing the MSM community. They explained that it had facilitated supportive solidarity and resulted in creation of safe spaces for previously very isolated men to befriend and communicate one another in a country which remained quite hostile to gay men. These safe spaces enabled MSM to express themselves freely without fear of discrimination or violence. The approach also supported the development of organizations which provided material and legal support to MSM.

*“Before [this NGO], we never used to have any form of support systems. But these days we come here if we feel lonely and mistreated and we can talk to people of like-mind. The offices here have provided comfort to most of us. That is why I spend a lot of time here. It is important in life to be with people who will understand you and treat you as a human being who deserves equal treatment and this is what this office does to me… There are avenues that you can benefit from if you feel you have been discriminated against. They have their own legal aid officers who can help you with some legal advice.”*

*-MSM FGD participant 3, early 30’s, employed*

MSM respondents also cited limitations of a rights-based approache. However, when noting the approach’s weakness, they framed their comments as suggestions for improvement, rather than implying a rejection of a rights-based approach overall. Their critical comments therefore contrast with those of many women and youth in our study. For example, rather than questioning the importance or appropriateness of focusing on human rights in HIV prevention, MSM respondents emphasized the need to first bolster the Zambian legal system to protect rights claims. They also noted the need for additional funding if human rights campaigns and organizations were to yield optimal benefits.

*“I feel Government does not offer much protection. Look at our systems, such as health and legal system. We do not have very strong legislation that protects people who identify themselves as gay or lesbian. The state has neglected us and I think they need to do more.”*

-*MSM FGD participant 6, mid-20’s, employed*

*“[The NGO] is doing a lot of good work… but they face some challenges. There is lack of adequate financing, because I know that with more money, we could have wider coverage, even in rural areas where people are more conservative.”*

*-MSM FGD participant 3, early 30’s, employed*

Some were critical of the ways the approach was implemented, saying it was too confrontational. As with women and youth, they identified ways in which it conflicted with local cultural and religious values.

*“Sometimes I think the resistance is due to the approach we use. We seem to be pushing the idea on the faces of the people too hard. It’s like we are forcing them to accept our views you know, much like they are doing themselves, making us accept theirs. We need a different approach I think. You and me both know that people here are too religious and they love their culture; they think our acts are unZambian, and so, the more we force them to accept something they do not agree with in the first place just makes things worse. I feel this is not the time to force people to accept the rights of homosexuals. I feel we first need to construct a narrative that will make these people empathetic. Show them the other side of a story in a very humble way, so that they begin to see things not as an attempt to fight their culture and religion, but rather we need to appeal to their sense of compassion and empathy in order for them to really get on board. We cannot continue to use this approach in the manner that it is being used in kuBazungu (Western countries)”*

*-MSM FGD participant 1, mid-30’s, employed*

However, rather than a rejection of the human rights approach, their views were all suggestions around how best to package and support the concept to make it more culturally and religiously sensitive to social relations in Zambia.

## Discussion

Zambian NGO employees viewed rights as important for addressing the roots of the HIV epidemic and appreciated the potential for human rights approaches to leverage critical resources from western development agencies. Yet, they also described how the lack of flexibility on the part of donor agencies regarding activity implementation and use of funds hampered their ability to deliver acceptable and relevant programs in the communities they sought to serve. These views were echoed by each of the three different client groups in group-specific ways. Women and youth widely criticized the human rights approach for conflicting with deeply held cultural and religious values and failing to account for the economic and social realities of their everyday lives. They described the concept of asserting their rights by challenging their husbands and fathers as ludicrous and dangerous. Even if the concept of human rights provided some basis for challenging harmful practices and abuse, for most women this did little to outweigh potential threats to social structures and relationships on which they relied for survival and identity. Similarly, although youth noted that it allowed for greater participation than previous HIV prevention efforts, overall they saw little potential benefit from rights that did nothing to further their goals of obtaining an education and employment and escaping poverty.

However, looking across all three groups, our findings also illustrate the diversity in the ways that the human rights approach is represented. Notably, it garnered widespread support from MSM, even as they noted ways in which its confrontational nature conflicted with the local culture. Given that our MSM respondents already felt deeply excluded from society, the potential for a rights-based approach to disrupt traditional social structures and cultural values posed little threat. Any potential threat was vastly outweighed by the positive potential to help them leverage support and challenge discrimination.

The HIV epidemic is driven by a complex mix of material and symbolic factors [43, 44]. The diverse social representations evident in our findings demonstrate ways in which the human rights approach fails to account for how deeply intertwined these two dimensions are. The rights-based approach seeks to tackle the symbolic drivers of the epidemic—its undeniable roots in cultural and religious systems of discrimination against women, youth, and sexual minorities [3]. Yet there are several ways in which this approach contradicts many of our informants’ own understandings of their needs and priorities. First, youth and women often see material issues as their main life challenges, particularly poverty and unemployment. Second, symbolic drivers, such as vast power inequalities due to traditional hierarchies of authority and defined social roles, often correlate closely with relationships that carry obligations of much needed material support.

Our findings regarding the views of women program beneficiaries concur with the conclusions of Campbell and Nair [36]—for women in settings of poverty and inequality, religious and cultural views of gender relations serve as vital economic protections. Traditionally mandated relationships with men are often the only source of economic survival available to women and their children. In contexts where men control access to land and have privileged access to scarce employment opportunities, uneducated and unskilled women would be destitute without the economic support of men who may be oppressive or abusive [15, 45]. The human rights challenge to gender-unequal worldviews and practices underestimates their vital role in women’s survival and fails to provide alternative survival strategies for women [36]. Moreover, as Mannell describes in her research on gender mainstreaming efforts within HIV-prevention [37, 46], notions of gender equality as envisaged by funding organizations may be viewed as a neo-colonial imposition of Western ideas. Several of our respondents expressed this sentiment, calling for locally-informed approaches to HIV prevention that fit the reality and needs of Zambian women. Our respondents’ comments also indicate skepticism of the NGO workers that champion human rights, who they describe as ‘unmarried’ or ‘divorced,’ with these two words carrying heavy connotations of difference from themselves. Self-sufficient single women represent a small minority of Zambian women who have managed to make lives independently of traditional cultural and religious relationships with men [14]. The option of being unmarried or divorced falls out of the range of possibility for most women.

Youth also set heavy store by traditional and religious world views and practices. They too rely on traditional family relationships as survival strategies. These relationships are critical in relation to the economic base of traditional family life, but also in relation to the comfort and direction and sense of belongingness and emotional sustenance they derive from them. Mostly uneducated and unskilled, the modern world and its associated worldviews does not seem accessible to them. The new ways of thinking that are implicit in human rights represent a world that they don’t see themselves as having access to. In its absence, they rely upon familiar worldviews and relationships. Many youth also viewed the emphasis on human rights as out-of-touch with their priorities and tangential to the structural factors that they described as the true causes of HIV vulnerability. Evoking the arguments of human rights approach critics like Farmer [31] and Englund [32], several youth respondents eloquently expressed their frustration with HIV-prevention efforts that advocated for awareness of rights while neglecting issues like poverty and unemployment.

In contrast to women and youth, MSM have little to gain from and often suffer greatly due to traditional religious and cultural norms. In Zambian society they are outcasts in a way that women and youth are not [23]. They are therefore the most positive about the rights-based approach as it has the most to offer them, given the context of extreme discrimination in which they live. Furthermore, particularly for those who do not disclose their sexual identity, their status as adult men affords them far greater control over access to material resources than women and youth in Zambian society. Thus while our other respondents viewed rights as tangential compared to issues of poverty and joblessness, MSM spoke far less about material concerns and instead emphasized the need for symbolic recognition of their equality. And yet even they, who stood to gain the most from a rights-based approach, felt that in its current form it is too confrontational to worldviews that Zambians hold dear.

There is no doubt that advocates of the human rights approach are right in pointing to inequalities and discrimination perpetuated by traditional values as a problem driving AIDS. However, currently the way in which they are imposing the approach onto local Zambians does not always resonate with the realities of those they are trying to serve. It also fails to take account of how deeply these problematic cultural norms are intertwined with many people’s economic survival strategies—particularly women and youth. Finally, it neglects the ways in which ordinary, unskilled, and uneducated people feel distanced from the ‘modern world’ which they perceive the human rights world to be part of.

## Conclusion

Against the background of disappointing outcomes of ABC approaches that dominated previous HIV-prevention efforts, human rights approaches have been widely mooted as the conceptual frame of choice by international funders and their associated NGOs in many Sub-Saharan Africa settings. Whilst the authors support the approach in principle, our findings highlight that it did not always resonate with members of key vulnerable groups. Social representations theory proved to be a useful analytical perspective for guiding exploration of our participants’ nuanced understandings of human rights. Importantly, the social representations lens served as a heuristic for respecting the contested and evolving nature of social knowledge by drawing our attention to the contrasts and commonalities within and between respondent groups. Rather than definitively establishing human rights approaches as an incontestable good, as they are often depicted in policy [34], or as useless because they ignore cultural and economic context as critics assert in the literature [32, 33], our findings instead indicate the diversity in how such approaches are locally viewed by vulnerable groups and the complexity of factors that influence ways in which they are represented.

Much research remains to be done on the generalizability of our findings to other settings in Sub-Saharan Africa, and to other Zambian populations such as those living in rural areas. Such studies would help to generate a more complete understanding of human rights within broader Zambian, and African, cultural-religious contexts. However, we argue that this study provides grounds for caution in assuming the universal resonance of a human rights approach. Overall, our findings point definitively to the folly of taking a one-size-fits-all approach to HIV-prevention and suggests that rigid donor agency stipulations regarding the activities that they fund may undermine the local expertise of implementing NGOs. It indicates the need for careful dialog between the designers of public health programs and their target groups in order to tailor HIV reduction strategies that take greater account of the closely intertwined nature of the material and symbolic in shaping peoples’ everyday lives and realities, and to identify enhanced opportunities for affected groups to develop new and more health-enhancing ways of ‘being, seeing, and doing.’

## Abbreviations

ABC: Abstain Be-Faithful and Condomize
AIDS: Acquired Immune Deficiency Syndrome
FGD: Focused Group Discussion
HIV: Human Immunodeficiency Virus
MSM: Men who have Sex with Men
NGO: Non-Governmental Organization

## Footnotes

1 The first author collected all data; the second author collaborated with the first author on analysis and interpretation of findings and drafting the manuscript; the third author guided development of the study concept, supervised data collection and analysis, and provided input throughout the manuscript writing process.

2 “*Kupyana*,” or “sexual cleansing,” refers to a traditional Zambian practice requiring a widow to have sex with a brother or cousin of her deceased husband in order to be cleansed of his spirit. Implication of this practice in the transmission of HIV has resulted in emergence of alternatives to *kupyana* and a decrease in its prevalence [39].
